# Supplementary material for: Nosustrophine: An Epinutraceutical Bioproduct with Effects on DNA Methylation, Histone Acetylation and Sirtuin Expression in Alzheimer’s Disease
Source: Pharmaceutics. 2022 Nov 12;14(11):2447. doi: 10.3390/pharmaceutics14112447 (PMC9698419; doi:10.3390/pharmaceutics14112447)
Supplement: Supplementary file 1 [file pharmaceutics-14-02447-s001.zip › Figure S1.pptx]

## Slide 1
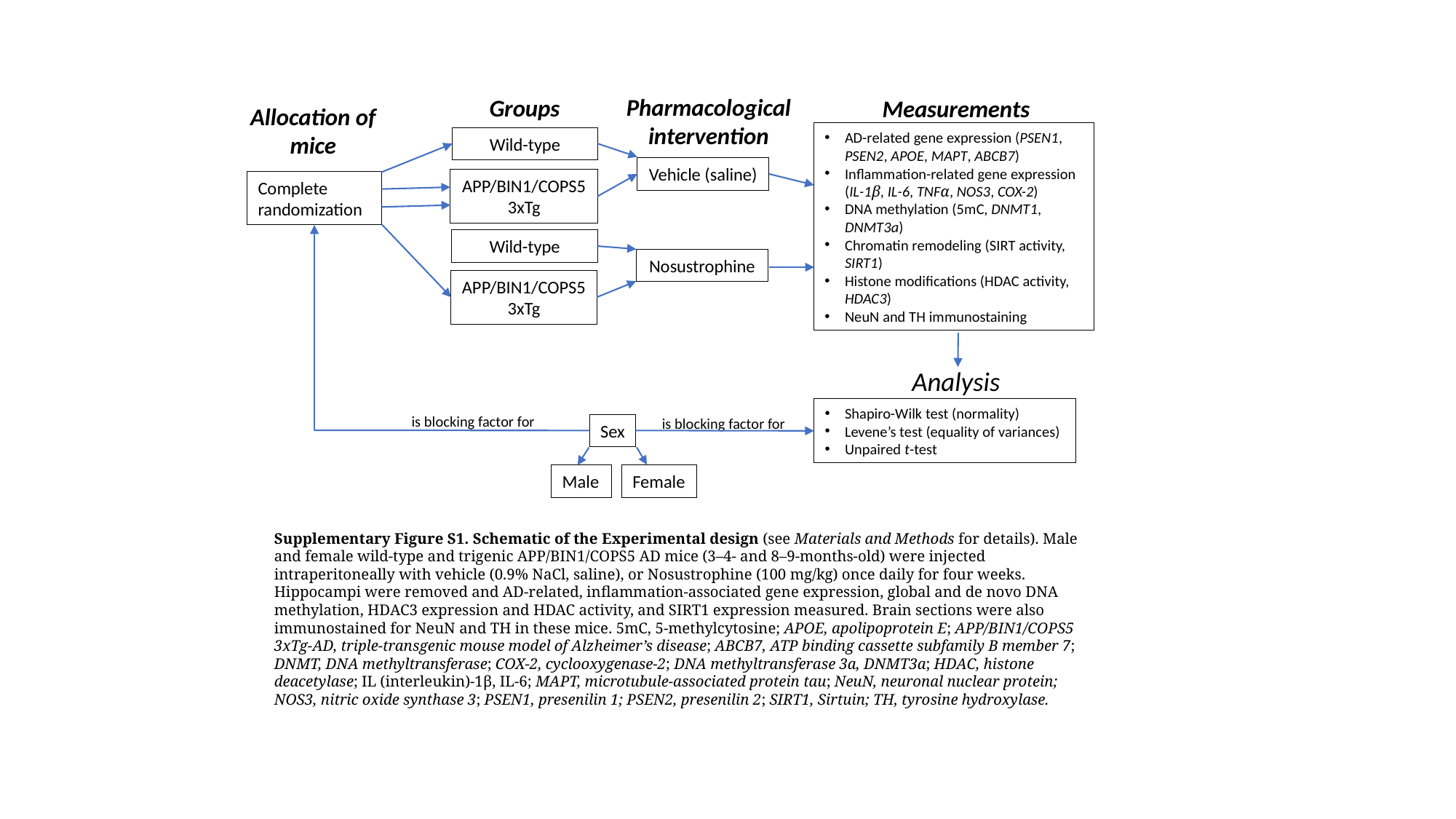

Pharmacological intervention
Groups
Measurements
Allocation of mice
AD-related gene expression (PSEN1, PSEN2, APOE, MAPT, ABCB7)
Inflammation-related gene expression (IL-1β, IL-6, TNFα, NOS3, COX-2)
DNA methylation (5mC, DNMT1, DNMT3a)
Chromatin remodeling (SIRT activity, SIRT1)
Histone modifications (HDAC activity, HDAC3)
NeuN and TH immunostaining
Wild-type
Vehicle (saline)
APP/BIN1/COPS5
3xTg
Complete randomization
Wild-type
Nosustrophine
APP/BIN1/COPS5
3xTg
Analysis
Shapiro-Wilk test (normality)
Levene’s test (equality of variances)
Unpaired t-test
is blocking factor for
is blocking factor for
Sex
Male
Female
Supplementary Figure S1. Schematic of the Experimental design (see Materials and Methods for details). Male and female wild-type and trigenic APP/BIN1/COPS5 AD mice (3–4- and 8–9-months-old) were injected intraperitoneally with vehicle (0.9% NaCl, saline), or Nosustrophine (100 mg/kg) once daily for four weeks. Hippocampi were removed and AD-related, inflammation-associated gene expression, global and de novo DNA methylation, HDAC3 expression and HDAC activity, and SIRT1 expression measured. Brain sections were also immunostained for NeuN and TH in these mice. 5mC, 5-methylcytosine; APOE, apolipoprotein E; APP/BIN1/COPS5 3xTg-AD, triple-transgenic mouse model of Alzheimer’s disease; ABCB7, ATP binding cassette subfamily B member 7; DNMT, DNA methyltransferase; COX-2, cyclooxygenase-2; DNA methyltransferase 3a, DNMT3a; HDAC, histone deacetylase; IL (interleukin)-1β, IL-6; MAPT, microtubule-associated protein tau; NeuN, neuronal nuclear protein; NOS3, nitric oxide synthase 3; PSEN1, presenilin 1; PSEN2, presenilin 2; SIRT1, Sirtuin; TH, tyrosine hydroxylase.
