# Supplementary material for: Nosustrophine: An Epinutraceutical Bioproduct with Effects on DNA Methylation, Histone Acetylation and Sirtuin Expression in Alzheimer’s Disease
Source: Pharmaceutics. 2022 Nov 12;14(11):2447. doi: 10.3390/pharmaceutics14112447 (PMC9698419; doi:10.3390/pharmaceutics14112447)
Supplement: Supplementary file 1 [file pharmaceutics-14-02447-s001.zip › Figure S2.pptx]

## Slide 1
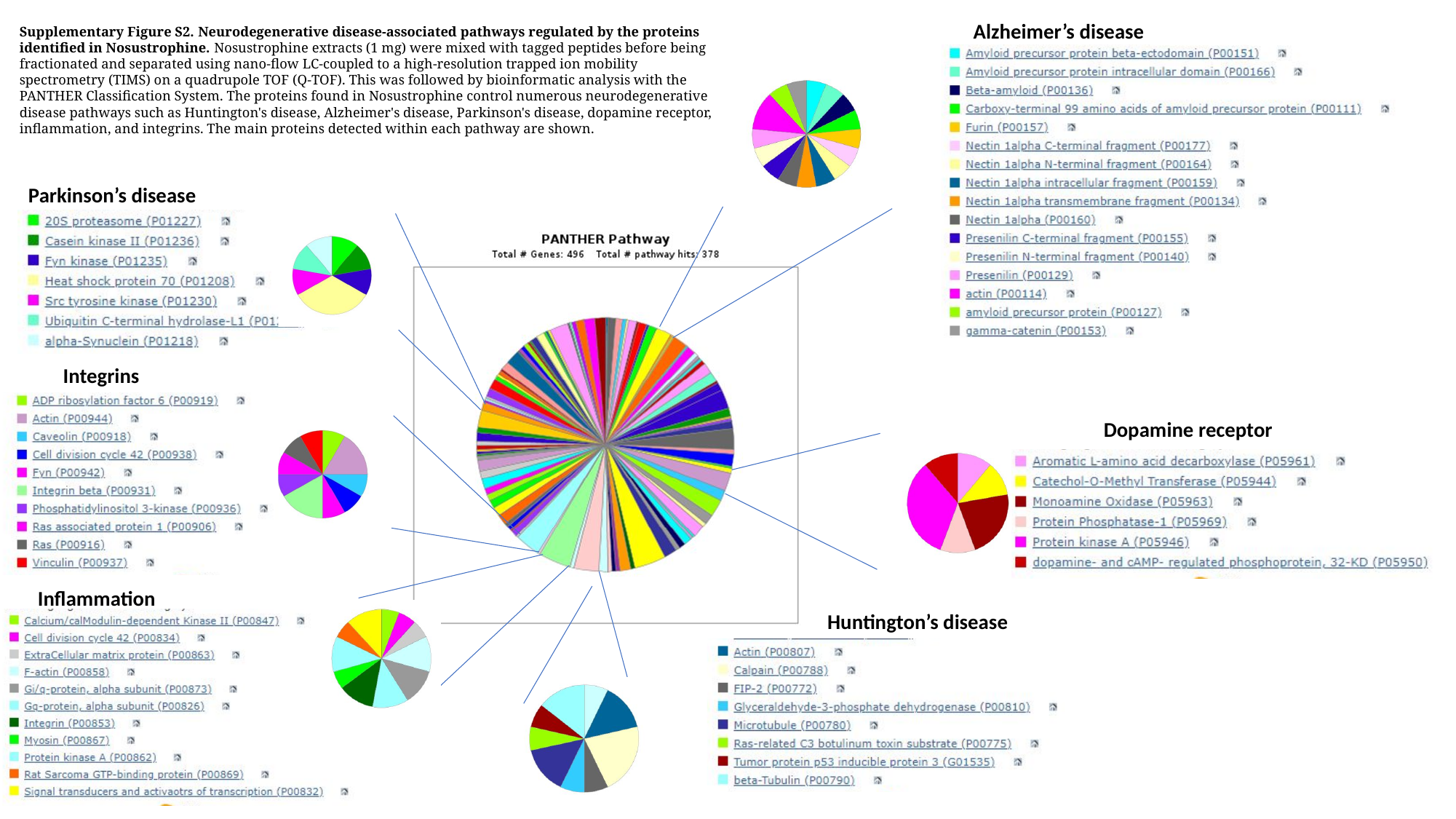

Alzheimer’s disease
Supplementary Figure S2. Neurodegenerative disease-associated pathways regulated by the proteins identified in Nosustrophine. Nosustrophine extracts (1 mg) were mixed with tagged peptides before being fractionated and separated using nano-flow LC-coupled to a high-resolution trapped ion mobility spectrometry (TIMS) on a quadrupole TOF (Q-TOF). This was followed by bioinformatic analysis with the PANTHER Classification System. The proteins found in Nosustrophine control numerous neurodegenerative disease pathways such as Huntington's disease, Alzheimer's disease, Parkinson's disease, dopamine receptor, inflammation, and integrins. The main proteins detected within each pathway are shown.
Parkinson’s disease
Integrins
Dopamine receptor
Inflammation
Huntington’s disease
