# Supplementary material for: Nosustrophine: An Epinutraceutical Bioproduct with Effects on DNA Methylation, Histone Acetylation and Sirtuin Expression in Alzheimer’s Disease
Source: Pharmaceutics. 2022 Nov 12;14(11):2447. doi: 10.3390/pharmaceutics14112447 (PMC9698419; doi:10.3390/pharmaceutics14112447)
Supplement: Supplementary file 1 [file pharmaceutics-14-02447-s001.zip › full blots.pptx]

## Slide 1
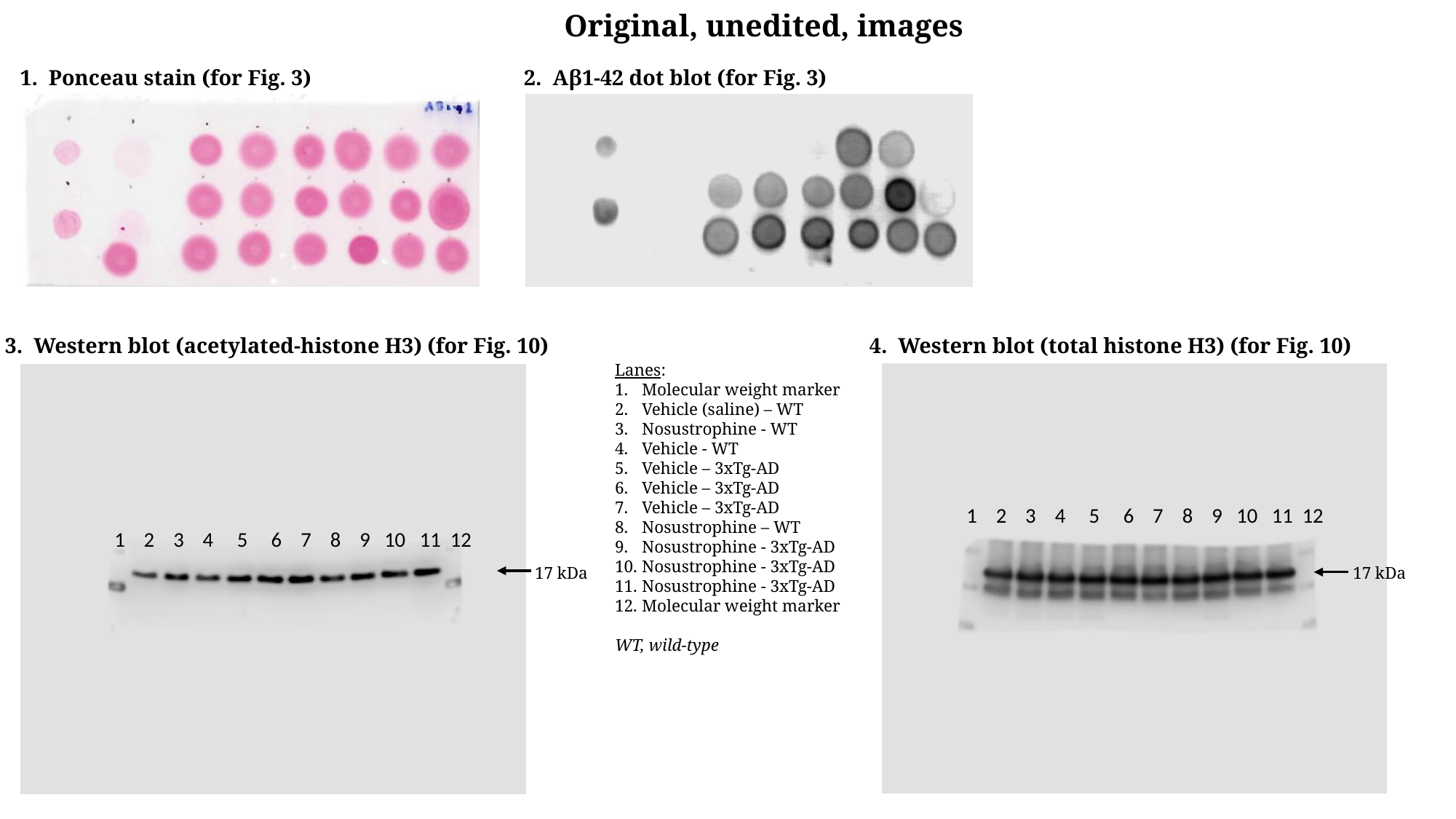

Original, unedited, images
1. Ponceau stain (for Fig. 3)
2. Aβ1-42 dot blot (for Fig. 3)
4. Western blot (total histone H3) (for Fig. 10)
1 2 3 4 5 6 7 8 9 10 11 12
 17 kDa
3. Western blot (acetylated-histone H3) (for Fig. 10)
1 2 3 4 5 6 7 8 9 10 11 12
 17 kDa
Lanes:
Molecular weight marker
Vehicle (saline) – WT
Nosustrophine - WT
Vehicle - WT
Vehicle – 3xTg-AD
Vehicle – 3xTg-AD
Vehicle – 3xTg-AD
Nosustrophine – WT
Nosustrophine - 3xTg-AD
Nosustrophine - 3xTg-AD
Nosustrophine - 3xTg-AD
Molecular weight marker
WT, wild-type
