# Supplementary material for: Nosustrophine: An Epinutraceutical Bioproduct with Effects on DNA Methylation, Histone Acetylation and Sirtuin Expression in Alzheimer’s Disease
Source: Pharmaceutics. 2022 Nov 12;14(11):2447. doi: 10.3390/pharmaceutics14112447 (PMC9698419; doi:10.3390/pharmaceutics14112447)
Supplement: Supplementary file 1 [file pharmaceutics-14-02447-s001.zip › Supplementary Tables.pdf]

Supplementary Table S2. List of primers used for genotyping APP/BIN1/COPS5 3xTg-AD mice.

Primer	Sequence
APP forward	AGG ACT GAC CAC TCG ACC AG
APP reverse	CGG GGG TCT AGT TCT GCA T
BIN/COPS5 forward	GAC TAC AAA GAC CAT GAC GGT
BIN reverse	CAG GTT AGT TTG AGC TAC GAG
COPS5 reverse	CCA CCC GAT TGC ATT TTC AAG
IL-2 forward	CTA GGC CAC AGA ATT GAA AGA TCT
IL-2 reverse	GTA GGT GGA AAT TCT AGC ATC ATC C

Supplementary Table S3. PCR conditions for genotyping APP/BIN1/COPS5 3xTg-A mice.

	Temperature	Time	Cycles
***APP* PCR**			
Denaturation	93 °C	3 min	1
Denaturation	93 °C	15 sec	40
Annealing	56 °C	30 sec	
Extension	68 °C	1 min	1
***BIN/COPS5* PCR**			
Denaturation	98 °C	30 sec	1
Denaturation	98 °C	5 sec	40
Annealing	52 °C	5 sec	
Extension	72 °C	15 sec	1
Extension	72 °C	1 min	1

Supplementary Table S4. Mice experimental design.

Group	Treatment	N	Genotype	Age (months)
A	Saline	4	Wild-type	3–4
B	Nosustrophine	4	Wild-type	3–4
C	Saline	4	APP/BIN1/COPS5	3–4
D	Nosustrophine	4	APP/BIN1/COPS5	3–4
E	Saline	4	Wild-type	8–9
F	Nosustrophine	4	Wild-type	8–9
G	Saline	4	APP/BIN1/COPS5	8–9
H	Nosustrophine	4	APP/BIN1/COPS5	8–9

Suppl. Table 5. List of primary antibodies used for immunohistochemistry, dot- and Western blots.

Antibody	Dilution	Species	Clonality	Supplier	Product number
NeuN	1:1000	Mouse	Monoclonal	Millipore	MAB-377
TH	1:1000	Mouse	Monoclonal	Millipore	IHCR1005-6
Aβ1-42	1:1000	Mouse	Monoclonal	Millipore	05-831-I
Acetyl-histone H3 (Lys14)	1:500	Rabbit	Polyclonal	Millipore	07-353

Histone H3	1:500	Rabbit	Polyclonal	Thermo Fisher	PA5-16183

Supplementary Table S6. List of TaqMan probes.

GENE	ID
PSEN1	Mm05001104_m1
PSEN2	Mm00440405_m1
APOE	Mm0137193_g1
MAPT	Mm00521988_m1
ABCB7	Mm01235250_m1
NOS3	Mm0045217_m1
COX-2	Mm0329438_g1
TNFα	Mm0044258_m1
IL-6	Mm00446190_m1
IL-1β	Mm00434228M1
DNMT1	Mm0048309_m1
DNMT3a	Mm043281_m1
SIRT1	Mm0168521_m1
HDAC3	Mm0515816_m1
S18	Mm03929990_g1
